# Efficient CO_2_ absorption through wet and falling film membrane contactors: insights from modeling and simulation

**DOI:** 10.1038/s41598-023-38249-9

**Published:** 2023-07-07

**Authors:** Nayef Ghasem

**Affiliations:** Department of Chemical and Petroleum Engineering, United Arab Emirates University, PO Box 15551, Al Ain, UAE

**Keywords:** Environmental sciences, Engineering

## Abstract

The release of excessive carbon dioxide (CO_2_) into the atmosphere poses potential threats to the well-being of various species on Earth as it contributes to global working. Therefore, it is necessary to implement appropriate actions to moderate CO_2_ emissions. A hollow fiber membrane contactor is an emerging technology that combines the advantages of separation processes and chemical absorptions. This study investigates the efficacy of wet and falling film membrane contactors (FFMC) in enhancing CO_2_ absorption in a monoethanolamine (MEA) aqueous solution. By analyzing factors such as membrane surface area, gas flow rate, liquid inlet flow rates, gas–liquid contact time, and solvent loading, we examine the CO_2_ absorption process in both contactors. Our results reveal a clear advantage of FFMC, achieving an impressive 85% CO_2_ removal efficiency compared to 60% with wet membranes. We employ COMSOL Multiphysics 6.1 simulation software and finite element analysis to validate our findings, demonstrating a close agreement between predicted and experimental values, with an average relative error of approximately 4.3%. These findings highlight the significant promise of FFMC for applications in CO_2_ capture.

## Introduction

The mass transfer and hydrodynamic performance of separation devices used in gas absorption processes are influenced by several crucial factors. These factors include the gas–liquid contact area, mass transfer coefficients, and pressure drop^1^. Membrane contactors (MCs) gain attention in pilot and industrial scales, particularly for carbon capture^2^, reducing energy consumption and cost. Challenges include pore wetting, solvent selection, and fouling with potential solutions. The review highlights working principles, comparisons with gas separation, module designs, and commercial implementations^3–5^. They consist of a porous membrane that acts as a physical barrier between the gas and liquid phases, allowing selective transfer of gases while preventing the mixing of the two phases. This unique structure enables several advantages regarding mass transfer rates and process efficiency^6^. MCs offer a substantial interfacial area, promoting the efficient mass transfer and enabling the effective removal of specific impurities like CO_2_ and H_2_S from gas streams.

Furthermore, MCs demonstrate low-pressure drops, decreasing energy consumption and operating costs^7,8^. MCs have been explored to replace traditional packed columns in carbon capture and sulfur dioxide removal applications^9^. Overall, the use of MCs represents a promising approach for gas–liquid mass transfer in various industries, and further research is needed to optimize their performance and commercial viability^10–13^. Hydrophobic polymer materials such as polypropylene, polytetrafluoroethylene, and polyvinylidene fluoride are the most common hydrophobic membranes used in CO_2_ absorption processes^14^. Membrane contactors, which are made up of several hollow fibers assembled, have a large specific surface area, providing a high interface area for gas–liquid contact^15–18^. In the membrane absorption process using hydrophobic membranes, the gas to be absorbed first transfers from the bulk gas phase to the gas-membrane boundary and then diffuses through the membrane pores to the gas–liquid interface where the absorption occurs^19^. The overall mass transfer process involves three resistances in series: the gas phase resistance, the membrane resistance, and the liquid resistance. Ideally, the membrane pores are filled with gas and reject liquid permeation to ensure low mass transfer resistance. However, most polymeric membranes used in membrane absorption are prone to wetting over prolonged periods of operation, which can negatively impact their performance^20^.

Membrane wetting can occur for various reasons, including non-optimal material properties such as inadequate hydrophobicity, wrong pore size, low chemical resistance, and operational issues such as liquid evaporation and condensation^21^. As a result, wetting of membrane pores is a significant technical challenge for membrane contactors, as it can significantly increase mass transfer resistance^22,23^. While some efforts have been made to improve material hydrophobicity and increase permeability by using asymmetric membrane structures to reduce pore wetting, it is still an ongoing issue. Alternatively, absorption processes that do not rely on hydrophobic pore-based membranes may be considered^24^. Using a hydrophilic porous membrane for CO_2_ absorption into aqueous solutions in a falling liquid-film mode is a novel approach that shows promise^25^. In this mode, the feed liquid permeates through the hydrophilic porous membrane pores, forming a falling liquid film on the surface of the permeation side of the membrane due to gravity. The thin liquid film can contact the gas fluid in a co- or counter-current flow^26^. In theory, the falling liquid film should completely cover the surface of the permeate side of a hydrophilic porous membrane^27^. As a result, the falling liquid-film approach offers the benefits of a high gas–liquid contact area while eliminating mass transfer resistance in the pores, making it an attractive alternative to hydrophobic pore-based membrane absorption^28,29^. Ceramic membranes have excellent mechanical strength, chemical stability, and thermal stability, making them resistant to corrosive amine solutions used for CO_2_ absorption^21,30–32^. Overall, ceramic membranes are well-suited for the falling liquid-film CO_2_ absorption process involving aqueous amine solutions^33–36^.

Several mathematical models were developed to depict the transport of gas impurities such as CO_2_ and H_2_S within a solvent across a hollow fiber membrane contactor^14–18,31,37,38^. A comprehensive model was developed to describe the gas and liquid reactions and transport inside the contactor under wetting or non-wetting conditions^39^. To the author's knowledge, no models have been developed thus far to comprehensively describe mass transport in a falling film membrane contactor. This study specifically examined the modeling and simulation of CO_2_ absorption using wet and falling liquid-film membrane contactors. Porous ceramic hollow hydrophilic membranes were employed in contactors with an aqueous monoethanolamine (MEA) solution. The research thoroughly compared wet membranes and falling film membrane contactors while exploring different operating parameters.

## Model development

**Figure 1:**
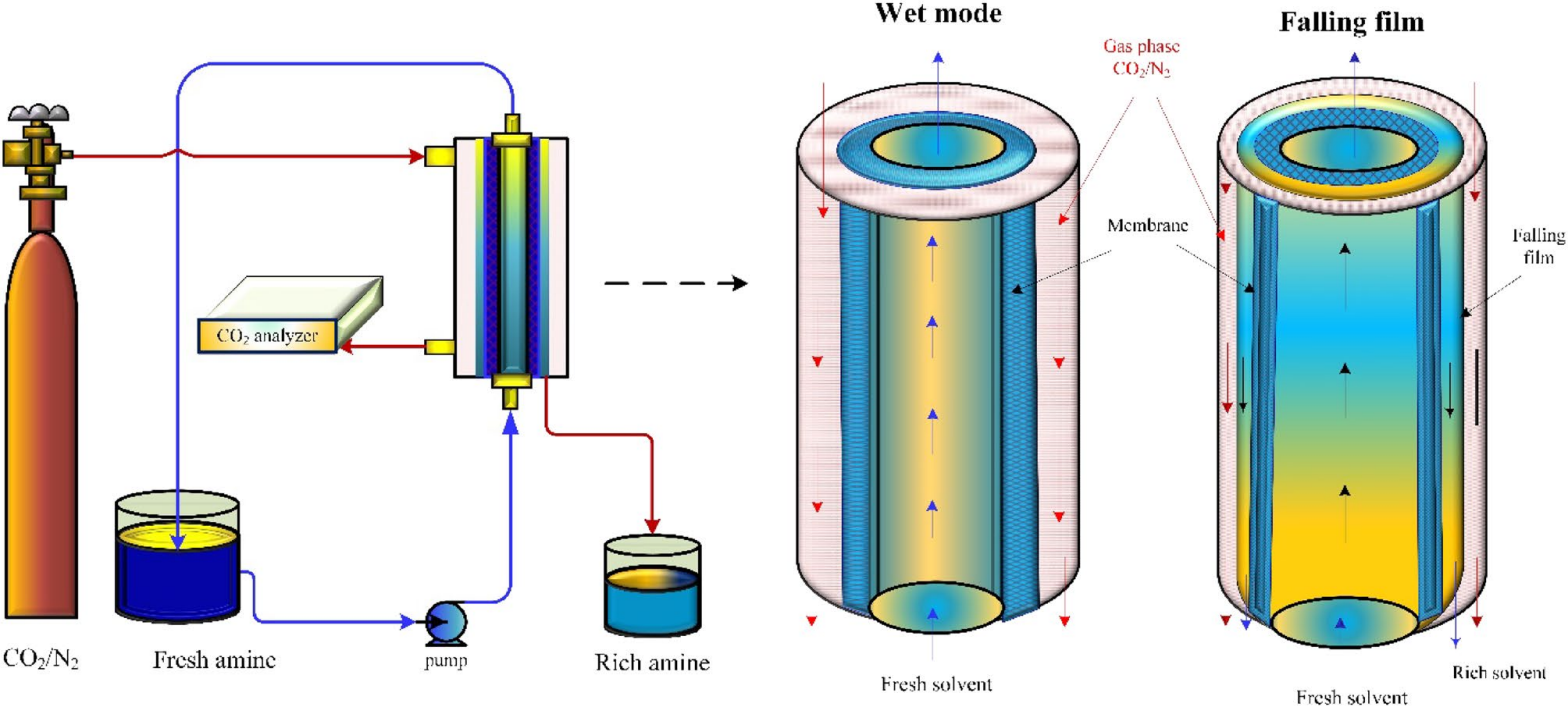
Schematic diagram describes solvent diffusion across membrane walls, forming a wet membrane (wet mode) and a thin falling film (falling film) to absorb CO_2_ from gas mixtures containing 10% CO_2_/N_2_. Figure generated using Microsoft Visio Professional 2016 (Microsoft.com).

The dynamic model that has been created depicts a two-dimensional simulation of the process of CO_2_ absorption from a gaseous mixture (CO_2_/N_2_) into an aqueous solvent of MEA, which occurs within a wet membrane mode, and a falling liquid film, membrane contactor (as shown in Fig. 1). The process involves solvent diffusion across the membrane walls, creating a thin solvent film. The solvent film is designed to selectively absorb CO_2_ from a gas mixture of 10% CO_2_; the remainder is N_2_.

The ceramic membrane used in this process is hydrophilic and includes an inner space called the lumen that the solvent travels through before reaching the membrane walls^40^. Once the solvent reaches the outside of the walls, it forms a thin film. The solvent film absorbs the CO_2_ from the inert ga moving in the opposite direction. As the inert gas flows, it creates a concentration gradient that causes the CO_2_ to be absorbed into the solvent as it passes near the solvent film. This method is commonly employed in various applications, including carbon capture, where the isolation of CO_2_ from other gases is necessary. By utilizing a ceramic membrane and solvent diffusion, it is possible to selectively extract CO_2_ from a gas stream, resulting in a cleaner gas product.

The falling film results from the diffusion of the amine solvent through the wet membrane porous to the gas side. In developing the transient model, the following assumptions are considered^38^:

1. Isothermal, steady state operating conditions.
2. The membrane is thoroughly wet.
3. Henry's law governs the gas–liquid equilibrium constant.
4. The gas is in ideal condition.
5. The velocity profile of the liquid in the tube and the inert gas in the shell are laminar.

Based on the steady-state assumption, the component material balance inside the tube (${C}_{i,t}$), taking into consideration the convective term, starts with velocity on the tube side (${V}_{z,t}$), diffusion term began with diffusion coefficient (${D}_{i,t}$), and reaction terms (${R}_{i}$) can be represented by Eq. (1):

1 $${D}_{i,t}\left(\frac{{\partial }^{2}{C}_{i,t}}{\partial {r}^{2}}+\frac{1}{r} \frac{\partial {C}_{i,t}}{\partial r}+\frac{{\partial }^{2}{C}_{i,t}}{\partial {z}^{2}}\right)+{R}_{i,t}-{V}_{z,t}\frac{\partial {C}_{i,t}}{\partial z}=0$$

The average velocity (${V}_{av}$) and the ratio of the variable radius ($r$) to the inner radius of the inner tube (${r}_{1}$) are used to represent the velocity profile on the tube side, as depicted by the following parabola. The Navier–Stocks equation is also applied, revealing notable variations in the velocity profile:

2 $${V}_{z,t}=2{V}_{av}\left[1-{\left(\frac{r}{{r}_{1}}\right)}^{2}\right]$$

Inside the membrane segment, due to the assumption of wetted mode, the membrane pores are full of solvent, and diffusion starts with ${D}_{i,m}$, and reactions are the only governing equations and the convective term is neglected, accordingly the concentration profile in the membrane pores (${C}_{i,m}$) represents the material component balance as follows:

3 $${D}_{i,m}\left(\frac{{\partial }^{2}{C}_{i,m}}{\partial {r}^{2}}+\frac{1}{r} \frac{\partial {C}_{i,m}}{\partial r}+\frac{{\partial }^{2}{C}_{i,m}}{\partial {z}^{2}}\right)+{R}_{i,m}=0$$

For the case solvent in the tube side and gas in the shell side, for a dry membrane (non-wetted), the diffusion coefficient of CO_2_ in the membrane pores (${D}_{i,m}$) is determined by porosity ($\epsilon$), tortuosity ($\tau$) of the porous membrane, and the diffusion of the component in the shell side (${D}_{i,s}$):

4 $${D}_{i,m}=\left[{D}_{i,s}\times \frac{\epsilon }{\tau }\right]$$

Based on the solvent flowing in the tube side, for a wet membrane (total wetting), the diffusion coefficient of CO_2_ in the membrane pores (${D}_{i,wm}$) is determined by porosity ($\epsilon$), tortuosity ($\tau$) of the porous membrane, and the diffusion of the component in the tube side (${D}_{i,t}$):

5 $${D}_{i,wm}=\left[{D}_{i,s}\times \frac{\epsilon }{\tau }\right]$$

The material balance governing the equation of existing components in the falling film is represented by the diffusion coefficient in the liquid film (${D}_{i,f}$), the concentration of the component on the film side (${C}_{i,f}$), and the reaction rate $({R}_{i,f})$, the film velocity can be neglected because it is prolonged:

6 $${D}_{i,f}\left(\frac{{\partial }^{2}{C}_{i,f}}{\partial {r}^{2}}+\frac{1}{r} \frac{\partial {C}_{i,f}}{\partial r}+\frac{{\partial }^{2}{C}_{i,f}}{\partial {z}^{2}}\right)+{R}_{i,f}=0$$

The concentration profile of carbon dioxide ($${C}_{i,s})$$ in the shell side is represented by the diffusion coefficient of CO_2_ on the shell side ($${D}_{i,s}$$), and the velocity profile on the shell side ($${V}_{z,s}$$), no reaction rate occurs on the side except in the falling film:

7 $${D}_{i,s}\left(\frac{{\partial }^{2}{C}_{i,s}}{\partial {r}^{2}}+\frac{1}{r} \frac{\partial {C}_{i,s}}{\partial r}+\frac{{\partial }^{2}{C}_{i,s}}{\partial {z}^{2}}\right)-{V}_{z,s}\frac{\partial {C}_{i,s}}{\partial z}=0$$

The Navier–Stokes equations regulate the flow of liquids and can be considered Newton's second law of motion for fluids. For a compressible Newtonian fluid, this results in the following:

8 $$\rho \left(\frac{\partial u}{\partial t}+u\cdot \nabla u\right)=-\nabla p+\nabla \cdot \left(\mu \left(\nabla u+{\left(\nabla u\right)}^{T}\right)-\frac{2}{3}\mu \left(\nabla \cdot u\right)I\right)+F$$

where "u" represents the velocity of the fluid, "p" represents the pressure of the fluid, "ρ" represents the density of the fluid, and "μ" represents the fluid's dynamic viscosity.

The separate terms refer to the inertial, pressure, viscous, and external forces acting on the fluid. The parameters used in the model development are shown in Table 1.

**Table 1:** Ceramic membrane speciation and feed conditions^27^.

Parameters	Value
Module length (cm)	30
Module diameter (cm)	0.18
Hollow fiber ID (mm)	12
Hollow fiber OD (mm)	8
Inlet concentration of CO_2_ (10%)	10
Inlet MEA concentration	5 M
CO_2_ loading (mol/mol)	0.2
Liquid feed rate (l/(m^2^ s)	0.2
Gas feed rate (mol/(m^2^ s)	1.3

The physical and chemical properties used in the modeling are shown in Table 2. The porosity of a hollow fiber ceramic membrane can vary depending on the specific membrane and its intended use. However, in general, the porosity of a hollow fiber ceramic membrane can range from around 30 to 50%^41^.

**Table 2:** Physical and chemical properties used for modeling and simulation.

Parameters	value	References
${D}_{C{O}_{2},shell }({\mathrm{m}}^{2}/\mathrm{s})$	$1.8\times {10}^{-5}$	^42^
${D}_{C{O}_{2},mem }({\mathrm{m}}^{2}/\mathrm{s})$	${D}_{C{O}_{2},shell}(\epsilon /\tau )$	^38^
${D}_{C{O}_{2},mea }({\mathrm{m}}^{2}/\mathrm{s})$	$2.35\times {10}^{-6}\mathrm{exp}(-2119/T)$	^43^
Porosity ($\epsilon )$	0.50	^44^
Tortuosity ($\tau$)	${\left(2-\epsilon \right)}^{2}/\epsilon$	^45^
CO_2_ solubility in MEA (mol/mol)	0.8	^43^

When considering the reaction kinetics, it is possible to express it as a second-order reaction involving the first order of each reactant species. By utilizing the following formula, the reaction rate can be determined at various temperatures^46^:

9 $$r=\frac{\left({10}^{10.99-\frac{2152}{T}}\right)}{1000}\cdot \left[MEA\right]\cdot [C{O}_{2}]$$

**Figure 2:**
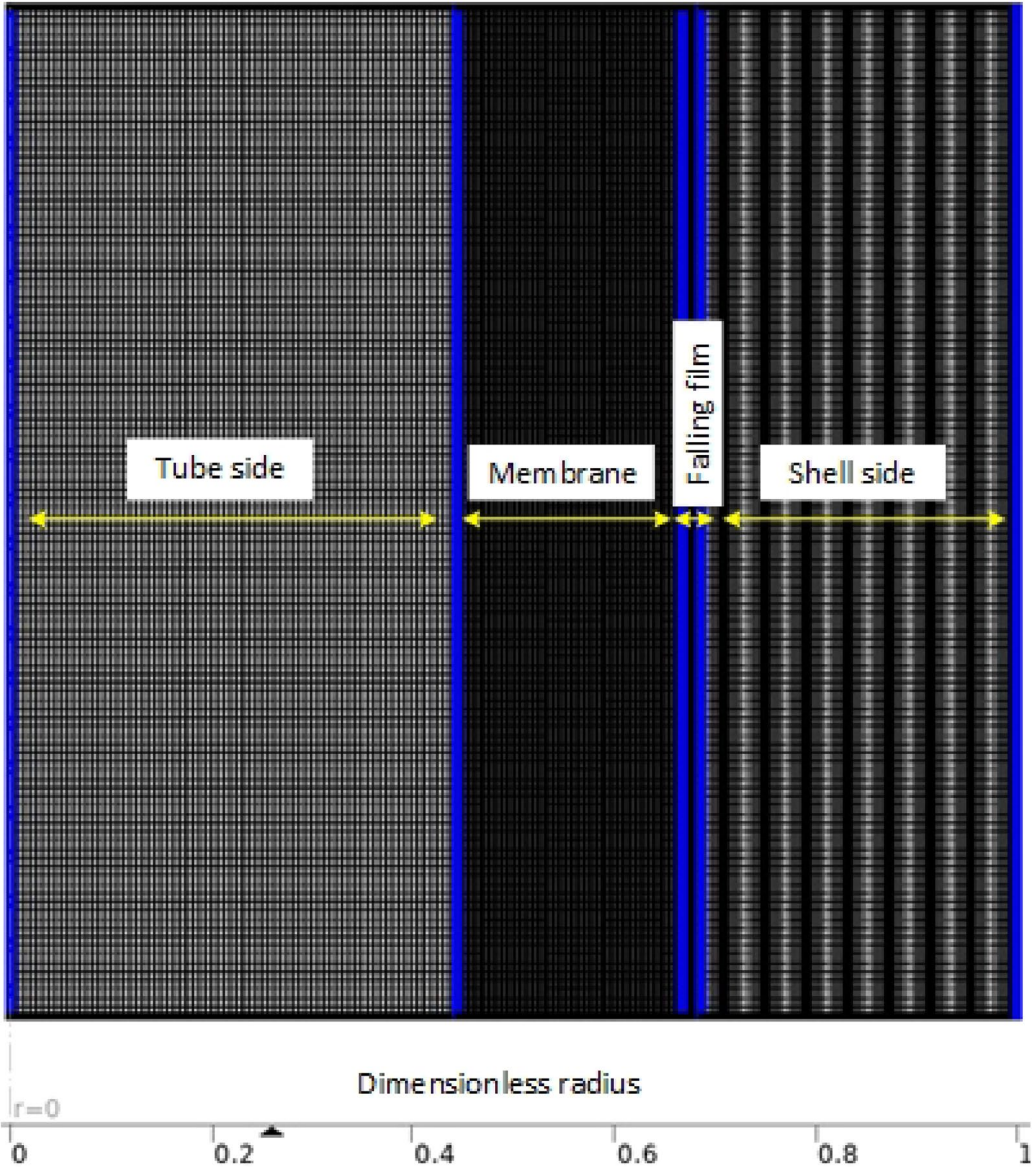
Optimum meshing of falling film membrane contactor domains generated using Comsol Multiphysics 6.1. Image generated using Comsol Multiphysics version 6.1 (comsol.com).

We used a computer program called COMSOL Multiphysics version 6.1^®^ to create a model of the transport of CO_2_ through a tube side, wet membrane, falling film, and shell side. The model used a Cartesian coordinate system to estimate the CO_2_ concentration at different points in the system. In order to discretize the computational domain, we are using structured multi-block grids, with the densest mesh being used in the region where the concentration gradient is largest and the flow phenomenon is most complex (presumably near the liquid film), and the loosest mesh being used in the shell side region where the velocity change is smallest and the flow phenomenon is simplest. After the meshing process is completed, we end up with a total of around 55,000 meshes. This information is shown in Fig. 2, which presumably provides a visual representation of the meshing scheme.

**Figure 3:**
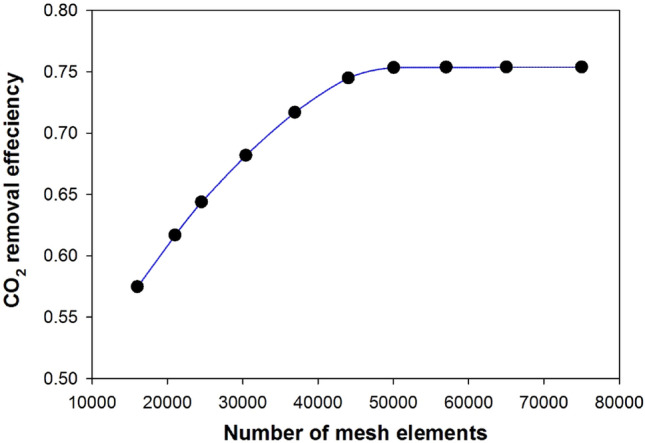
Simulation of CO_2_ removal fraction in the falling film membrane contactor conducted using COMSOL 6.1 software, considering the number of mesh elements used for discretization.

Figure 3 shows the mesh analysis approach used in the simulation and calculation of the CO_2_ removal fraction in the falling film membrane contactor. The CO_2_ removal efficiency does not change significantly when the number of mesh exceeds fifty thousand.

## Results and discussion

**Figure 4:**
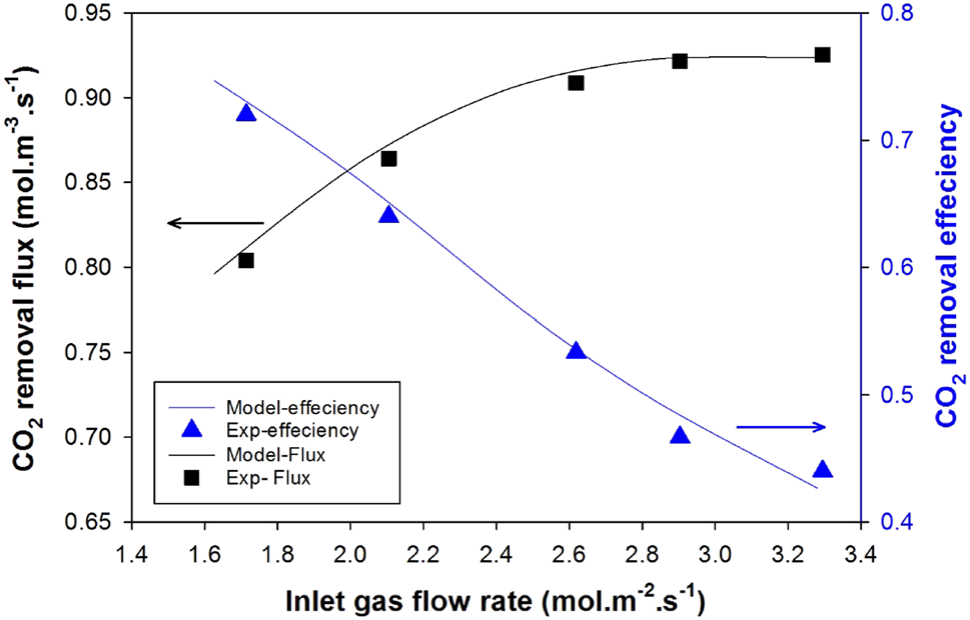
Comparing model predictions of CO_2_ removal flux and efficiency against gas flow rate in a falling film membrane contactor with the corresponding experimental data^27^.

Figure 4 compares mathematical model predictions with experimental data for CO_2_ removal efficiency and removal flux as a function of inlet gas feed rate. The results indicate that as the inert gas flow rate increases, the CO_2_ removal fraction decreases while the CO_2_ adsorption rate increases^47^. A higher gas flow rate reduces the amount of time CO_2_ spends in the membrane contactor, leading to a reduction in CO_2_ removal efficiency. On the other hand, a higher gas flow rate provides more CO_2_ for absorption, increases turbulence, and decreases the gas-side mass transfer resistance, contributing to an increase in the CO_2_ absorption rate. However, this increase slows as the gas flow rate rises because the proportion of resistance from gas phase mass transfer decreases, and the process becomes dominated by liquid-film control^23^. The model's predictions agree with the experimental data, demonstrating its reliability and making it a valuable tool for probing other parameters.

**Figure 5:**
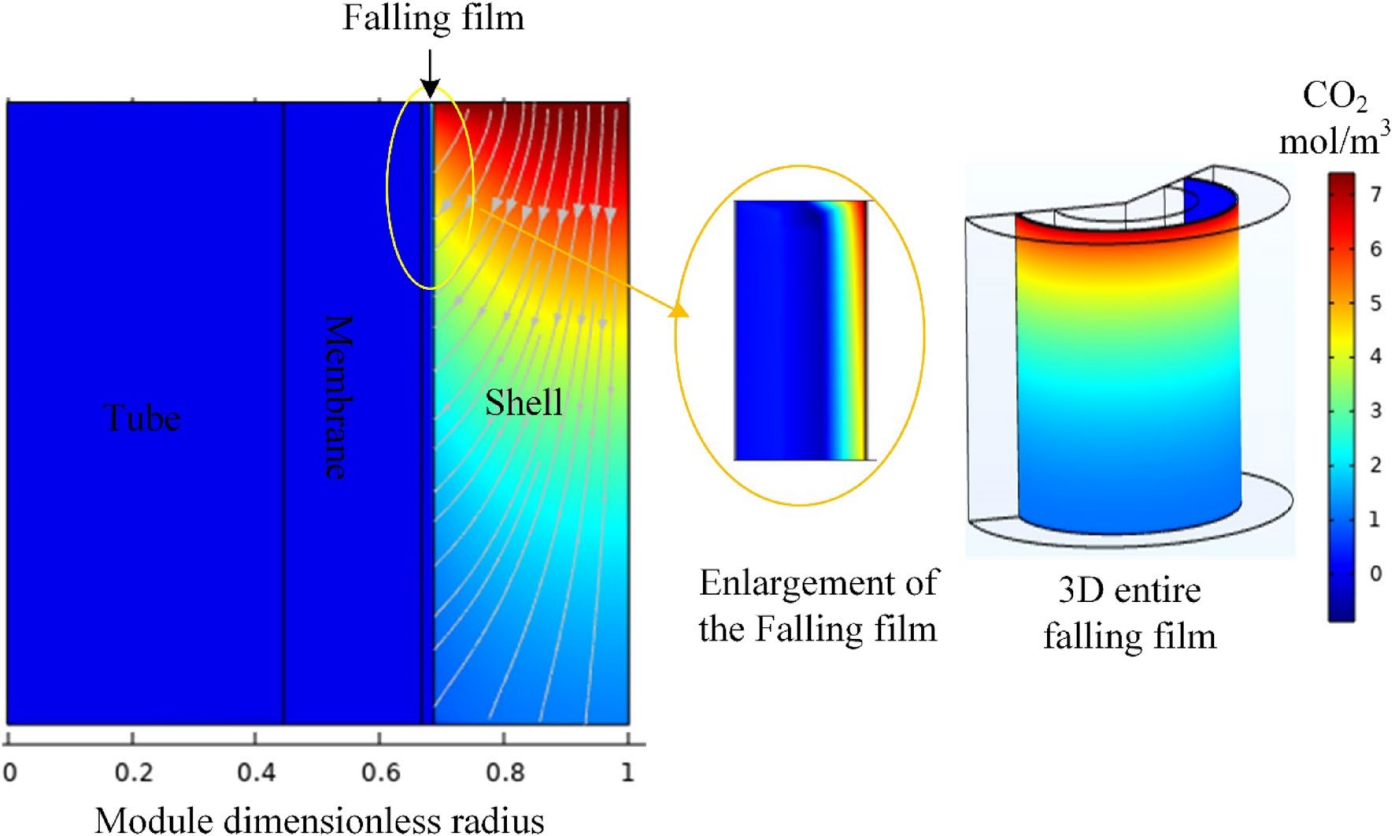
The surface plot of the CO_2_ concentration profile across the membrane module at a gas feed rate is 100 ml/min and a liquid feed rate of 60 ml/min. The image was generated using Comsol Multiphysics version 6.1 (comsol.com).

Figure 5 is a 2D surface plot that illustrates the changes in the concentration of CO_2_ across the membrane module, where the concentration of CO_2_ on the membrane shell side decreases due to absorption by the solvent film. This absorption mechanism is commonly used in gas separation processes, which creates a concentration gradient across the membrane, driving the passage of CO_2_ from the feed gas stream to the solvent. As a result, the concentration of CO_2_ in the feed stream decreases as it passes through the membrane module, while the concentration of CO_2_ in the permeate solvent stream increases^48^. The total flux arrows indicate the direction of CO_2_ transport through the shell side, eventually absorbed in the thin solvent film. In this case, the solvent film absorbs CO_2_, creating a concentration gradient across the falling liquid films. The concentration gradient created by the absorption of CO_2_ by the solvent film drives the passage of CO_2_ from the feed gas stream to the wetted membrane falling film^49^. However, CO_2_ concentration in the permeate stream is increasing, indicating that the solvent is capturing and transferring more CO_2_ through the wetted membrane film^50^.

**Figure 6:**
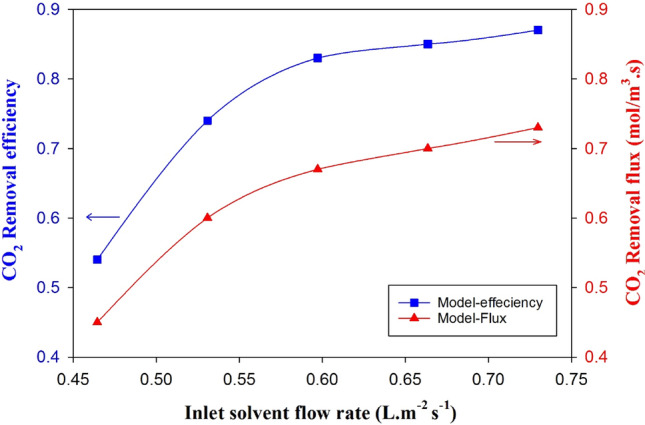
Effect of solvent feed rate on the absorption removal efficiency and removal flux of carbon dioxide from an inert gas (10% CO_2_/N_2_) in a falling film membrane contactor.

Figure 6 demonstrates that increasing the liquid flow rate improves the ability to absorb CO_2_ and absorption efficiency. This is due to two primary reasons: a higher liquid flow rate results in more active MEA molecules, enhancing absorption capacity and reaction kinetics. Secondly, the increased turbulence of the falling liquid film boosts the mass transfer coefficient, which is essential for liquid phase-controlled mass transfer. Nevertheless, avoiding excessively increasing the liquid flow rate is crucial since the absorption flux reaches a plateau^51^. The point at which the absorption flux reaches a plateau depends on the specific absorption process and the properties of the absorbent solution. It is essential to optimize the liquid flow rate to achieve the highest possible absorption efficiency without exceeding the point where the absorption flux plateaus^52^.

The typical concentration of monoethanolamine (MEA) in carbon dioxide (CO_2_) absorption processes can vary depending on the application and operating conditions. However, the typical range is around 20–30 weight percent (wt%) MEA^53^. Higher MEA concentrations can increase the solubility of CO_2_ in the solvent and improve the CO_2_ removal efficiency, but they also increase the energy consumption and cost associated with the regeneration process.

**Figure 7:**
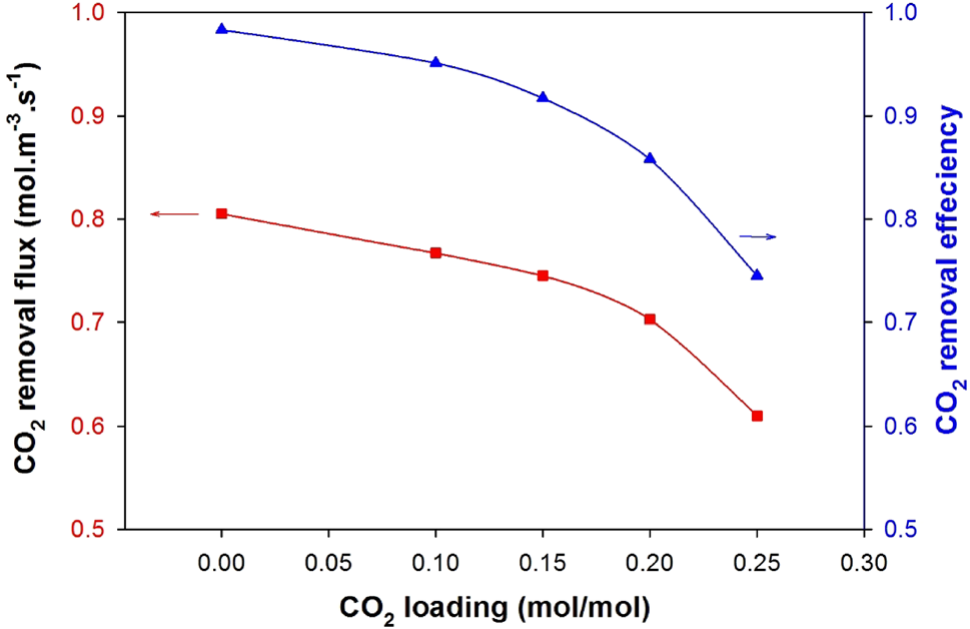
Effect of CO_2_ loading in the MEA aqueous solution on the CO_2_ fraction removal and removal flux for a falling film membrane contactor. Gas feed rate 100 ml/min.

Figure 7 shows that the effect of carbon dioxide (CO_2_) loading in aqueous monoethanolamine (MEA) on the CO_2_ fraction removal fraction and flux from simulated flue gas can be significant. MEA is commonly used as a solvent in chemical absorption processes for capturing CO_2_ from flue gas, biogas, and natural gas. As the CO_2_ loading in the MEA solution increases, the CO_2_ removal efficiency and flux typically decrease^54^. This is because when the concentration of CO_2_ increases, it becomes less soluble in the MEA solution, which reduces the driving force for CO_2_ absorption. Moreover, when there is a higher CO_2_ loading, solid particles, also known as "MEA salts", can form, lowering the CO_2_ removal efficiency and increasing the pressure drop in the absorber^55^. To maintain a high CO_2_ removal efficiency and flow rate, it is necessary to regularly regenerate the MEA solution to remove the absorbed CO_2_ and maintain a low CO_2_ concentration. Regenerating the MEA solution involves heating it to release the CO_2_, which is then typically compressed and stored for later use or permanent disposal. Controlling the CO_2_ loading in the MEA solution is essential for effective CO_2_ capture while minimizing the energy consumption and cost of the regeneration process when capturing CO_2_ from natural gas or flue gas^56^.

**Figure 8:**
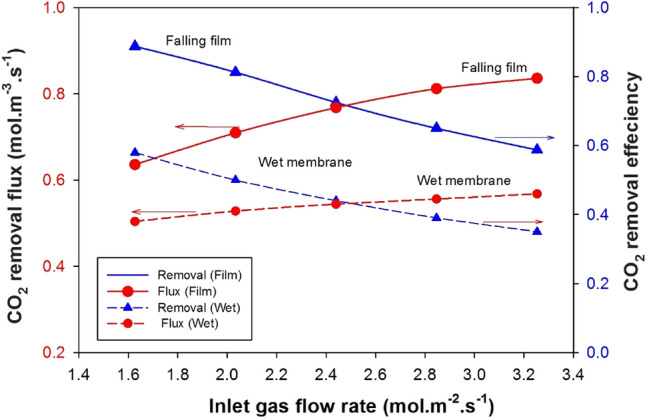
Comparison between falling fall and wet mode membranes for the effect of inlet gas flow rate on the CO_2_ removal efficiency and removal flux.

Figure 8 demonstrates how the gas feed rate affects the CO_2_ removal fraction and flux for the falling film and wet-membrane cases. In both cases, as the gas flow rate increases, the CO_2_ removal efficiency decreases because the gas has less time to dissolve the CO_2_ in the liquid film due to the reduced contact duration. Moreover, a higher gas flow rate can disrupt the liquid film, causing decreased contact between the gas and liquid phases, further reducing CO_2_ removal efficiency. In contrast, the rise in CO_2_ flux as the gas flow rate increases in a descending layer of MEA can be ascribed to heightened turbulence and improved mixing at higher gas flow rates. This augmented turbulence facilitates improved interaction between the gas and liquid phases, resulting in enhanced CO_2_ transfer from the gas to the liquid. Moreover, the increased gas flow rate leads to a thinner liquid film, reducing film resistance and further boosting the CO_2_ removal flux^57^. As a result, while the CO_2_ removal efficiency declines with higher gas flow rates, as explained earlier, the CO2 flux can increase. This is because the enhanced mass transfer and reduced film resistance associated with increased gas flow rates contribute to a higher rate of CO_2_ transfer^37^. In a wet membrane system, membrane thickness and gas permeation velocity may limit the absorption rate. In contrast, a falling film system has a higher surface area-to-volume ratio, resulting in a higher absorption rate^58^.

## Conclusions

Based on the simulation results, the falling film membrane system offers a higher CO_2_ absorption rate and removal flux than the wet membrane system. The falling film system offers an average CO_2_ removal efficiency of 85% with a removal flux of 0.82 mol/m^3^ s, while the wet membrane system achieves an average removal efficiency of 60% with a removal flux of 0.55 mol/m^3^ s. These quantitative findings indicate that the falling film presents a more optimal choice for achieving elevated levels of CO_2_ removal in specific applications. Good agreement was observed between experimental data and model predictions, with an average error of around 4%. The governing equations of the model have been successfully solved using COMSOL Multiphysics version 6.1. The findings demonstrated that higher gas flow rates could reduce the resistance to mass transfer on the gas side, resulting in an increase in both the removal flux and the reduction of removal efficiency. As the CO_2_ loading in MEA increases, it can lead to a decrease in both the removal efficiency and removal flux. The strong agreement observed between the mathematical model developed in this study and the experimental data demonstrates the reliability and suitability of the model for investigating the CO_2_ absorption process in MEA solutions using both wet and falling film membrane systems. Such investigations can expand our understanding of CO_2_ absorption processes and contribute to the development of more efficient and versatile carbon capture systems.

## Data Availability

All data generated or analyzed during this study are included in this published article.
