# Author Correction: Efficient CO_2_ absorption through wet and falling film membrane contactors: insights from modeling and simulation

**DOI:** 10.1038/s41598-023-39137-y

**Published:** 2023-07-24

**Authors:** Nayef Ghasem

**Affiliations:** Department of Chemical and Petroleum Engineering, United Arab Emirates University, PO Box 15551, Al Ain, UAE

Correction to: *Scientific Reports* https://doi.org/10.1038/s41598-023-38249-9, published online 07 July 2023

The original version of this Article contained an error in Reference 14, which was incorrectly given as:

Ghasem, N. & Al-Marzouqi, M. Modeling and experimental study of carbon dioxide absorption in a flat sheet membrane contactor. *J. Membr. Sci. Res.* **3**, 25 (2017).

The correct reference is listed below:

Ghasem, N. Modeling and simulation of CO_2_ absorption enhancement in hollow-fiber membrane contactors using CNT–water-based nanofluids. *J. Membr.* Sci. Res.** 5**, 295–302 (2019).

The original Article has been corrected.

